# Synergistic effects of mixing hybrid poplar and wheat straw biomass for bioconversion processes

**DOI:** 10.1186/s13068-015-0414-9

**Published:** 2015-12-24

**Authors:** Rodrigo Morales Vera, Renata Bura, Rick Gustafson

**Affiliations:** School of Environmental and Forest Sciences, University of Washington, Box 352100, Seattle, WA 98195-2100 USA

**Keywords:** Hybrid poplar, Wheat straw, Mixed biomass, Biomass blending, Steam pretreatment, Saccharification, Sugar yields, Biorefinery

## Abstract

**Background:**

Low cost of raw materials and good process yields are necessary for future lignocellulosic biomass biorefineries to be sustainable and profitable. A low cost feedstock will be diverse, changing as a function of seasonality and price and will most likely be available from multiple sources to the biorefinery. The efficacy of the bioconversion process using mixed biomass, however, has not been thoroughly investigated. Considering the seasonal availability of wheat straw and the year round availability of hybrid poplar in the Pacific Northwest, this study aims to determine the impact of mixing wheat straw and hybrid poplar biomass on the overall sugar production via steam pretreatment and enzymatic saccharification.

**Results:**

Steam pretreatment proved to be effective for processing different mixtures of hybrid poplar and wheat straw. Following SO_2_-catalyzed steam explosion pretreatment, on average 22 % more sugar monomers were recovered using mixed feedstock than either single biomass. Improved sugar recovery with mixtures of poplar and wheat straw continued through enzymatic hydrolysis. After steam pretreatment and saccharification, the mixtures showed 20 % higher sugar yields than that produced from hybrid poplar and wheat straw alone.

**Conclusions:**

Blending hybrid poplar and wheat straw resulted in more monomeric sugar recovery and less sugar degradation. This synergistic effect is attributable to interaction of hybrid poplar’s high acetic acid content and the presence of ash supplied by wheat straw. As a consequence on average 20 % more sugar was yielded by using the different biomass mixtures. Combining hybrid poplar and wheat straw enables sourcing of the lowest cost biomass, reduces seasonal dependency, and results in increasing biofuels and chemicals productivity in a cellulosic biorefinery.

**Electronic supplementary material:**

The online version of this article (doi:10.1186/s13068-015-0414-9) contains supplementary material, which is available to authorized users.

## Background

Good process yields are necessary for lignocellulosic biomass biorefineries to be profitable. Considerable research has been done to improve sugar yields from biomass feedstocks by chemically modifying raw materials using genetic engineering techniques [1, 2], developing novel pretreatment methods and reactor designs [3–5], and designing reactors for saccharification of high consistency solids [6]. However, there has been minimal research on pre-processing of lignocellulosic biomass as an alternative way to improve the overall biomass to sugar conversion.

Feedstock cost is a major determinant of the viability of commercial scale production of fuels and chemicals, contributing to 40–50 % of the operating costs in a lignocellulosic biomass-based biorefinery [7]. In addition, the low selling price of fuel products generated by a biorefinery does not allow the biomass conversion facility to purchase ‘pristine’ feedstocks composed of clean, homogeneous, and high-quality biomass [8]. Moreover, a consistent and stable supply of feedstocks will be required by the biorefineries to maintain the high throughput they require to be profitable. It is imperative to process the cheapest raw material available in a specific region to enable economically viable and sustainable processes of converting biomass to fuels and chemicals. These feedstocks will typically be diverse and will change as a function of time and price. They will most likely be available as a heterogeneous input stream to the biorefinery [9]. Consequently, a biomass processing facility must be able to convert diverse feedstock without significant penalties in overall performance, sugar yields, and fuel production. Currently, most bioconversion research has been carried out with high quality, relatively uniform raw materials, such as screened wood chips, while little attention has been paid to the efficiency of converting heterogeneous mixtures of feedstocks into fermentable sugars and fuels. Shi et al. [9] reported that ionic liquids can efficiently pretreat mixtures containing switchgrass, lodgepole pine, corn stover, and eucalyptus with no obvious negative impact on sugar yield. Yu and Chen [10] evaluated dilute acid, lime, and aqueous ammonia pretreatments for ethanol production using mixed feedstock. The mixture was composed of equal parts of wheat straw (WS), barley straw, hardwood, and softwood. The ethanol yield from mixed feedstock was similar to individual biomass samples for all pretreatment technologies evaluated. These findings indicate that mixed feedstocks may be a viable and valuable resource to consider when assessing biomass availability.

In the Pacific Northwest of the United States, there is 20,000 ha hybrid poplar (HP) [11] in production, with yields ranging from 6.9 to 19.1 metric tonne ha^−1^ year^−1^ [12] and approximately 1.2 million ha of planted wheat [13] producing more than 3 million dry tonne per year of WS [14, 15]. HP may be harvested year round, while WS is seasonally available. WS costs much less than HP ($24–50 [7, 16] vs. $77–105/dry tonne [12, 17]). These characteristics make HP and WS excellent potential raw materials for a lignocellulosic-based biorefinery in this region.

Despite the fact that several studies have successfully showed the advantages of processing HP and WS for the production of fuels and chemicals [18–22], there are no studies focused on the bioconversion of combined woody biomass and agricultural residues via steam explosion pretreatment and enzymatic hydrolysis. Thus, the combining of WS and HP for the production of sugars is an unexplored opportunity for maintaining the productivity and profitability of a biorefinery. In this regard, this research investigated the effect of using mixed feedstocks on bioconversion for sugar production. The goal of this study was to determine the impact of mixing WS and HP biomass on the overall sugar production via steam pretreatment and enzymatic saccharification, and to assess the robustness of steam explosion as a pretreatment for concurrently processing different combinations of HP and WS. Specifically, the objectives of this investigation were to assess the effect of mixing HP and WS on the following: (1) chemical properties of solid and liquid streams, and the sugar recovery after steam pretreatment, (2) enzymatic digestibility of solids, and (3) the overall sugar yields after steam explosion pretreatment and enzymatic hydrolysis.

## Results and discussion

In this study, HP, WS, and three mixtures with different combinations of both types of biomass (M1: 75 % HP, 25 % WS; M2: 50 % HP, 50 % WS and M3: 25 % HP, 75 % WS) were used to determine the impact of using mixed biomass on overall sugar yields following steam pretreatment and enzymatic hydrolysis. It is well know that HP and WS can be successfully converted to sugars via steam pretreatment and saccharification [18–22]; however, the impact of mixing these biomass types on overall sugar yields is unknown. We have characterized the unpretreated raw material, the liquids and solids after pretreatment and determined the overall sugar yield (kg/tonne of raw biomass) after saccharification to facilitate future techno-economic comparisons of using WS and HP mixtures.

### Raw material composition

The composition of the original untreated HP, WS, and the different mixtures in kg/tonne of raw material are presented in Table 1. The total polysaccharide content of all the biomass ranged from 525 to 558 kg/tonne. The compositional analysis showed statistically significant differences for most of the components found in HP and WS (*p* value <0.05) except galactan and total lignin. Arabinan, xylan, extractives, and ash contents increased from 2 to 20 kg/tonne, from 120 to 175 kg/tonne, from 61 to 94 kg/tonne, and from 5 to 43 kg/tonne, respectively, when WS was added to the samples. The contents of glucan, mannan, and acetic acid decreased from 415 to 326 kg/tonne, from 17 to 0 kg/tonne, and from 37 to 26 kg/tonne, respectively, when WS was supplemented to the mixtures. The presence of both acid-soluble lignin (ASL) and acid-insoluble lignin (AIL) was similar among the different samples, ranging from 235 to 245 kg/tonne. Statistically significant differences were found among the mixtures in terms of extractives and ash content ranging from 61 to 83 kg/tonne and from 15 to 34 kg/tonne, respectively (*p* value <0.05). The sugar composition of the HP and WS was similar to compositions observed by other investigators. However, differences were observed for WS lignin which was 7 % higher, and extractives content which was 6 % lower than values found by Ballesteros et al. [21].

**Table 1:** Compositional analysis of raw biomass (kg/tonne of raw material)

	HP	M1	M2	M3	WS
Arabinan	2 ± 0	8 ± 2	12 ± 1	18 ± 1	20 ± 1
Galactan	4 ± 0	4 ± 0	4 ± 1	4 ± 1	4 ± 1
Glucan	415 ± 15	366 ± 10	377 ± 14	364 ± 7	326 ± 14
Xylan	120 ± 10	152 ± 4	148 ± 2	153 ± 10	175 ± 2
Mannan	17 ± 1	15 ± 1	12 ± 1	7 ± 1	0 ± 0
Total sugars	558 ± 26	545 ± 17	553 ± 19	546 ± 20	525 ± 18
Acetate	37 ± 0	32 ± 1	30 ± 1	29 ± 3	26 ± 2
Total lignin	242 ± 4	238 ± 2	239 ± 4	245 ± 4	235 ± 9
Extractives	61 ± 2	61 ± 1	73 ± 4	83 ± 5	94 ± 5
Ash	5 ± 1	15 ± 0	25 ± 0	34 ± 0	43 ± 0

Total lignin: ASL + ASL

±Standard deviation are from triplicate measurements

*HP* hybrid poplar, *WS* wheat straw, *M1* 75 % HP, 25 % WS, *M2* 50 % HP, 50 % WS and *M3* 25 % HP, 75 % WS

HP and WS are made up of different types of hemicellulose. Glucuronoxylan has been identified as the main hemicellulose in poplar, whereas the most abundant hemicellulose constituents in WS—as well as in corn stover, rye, barley, oat, rice, and sorghum—are arabinoxylans [23, 24]. The arabinan content increases, therefore, when WS is added to poplar feedstock (Table 1). Glucose and xylose made up the majority of carbohydrates in the raw material, while arabinose, galactose, and mannose were present as minor sugars (Table 1).

### Solids composition after pretreatment

**Fig. 1:**
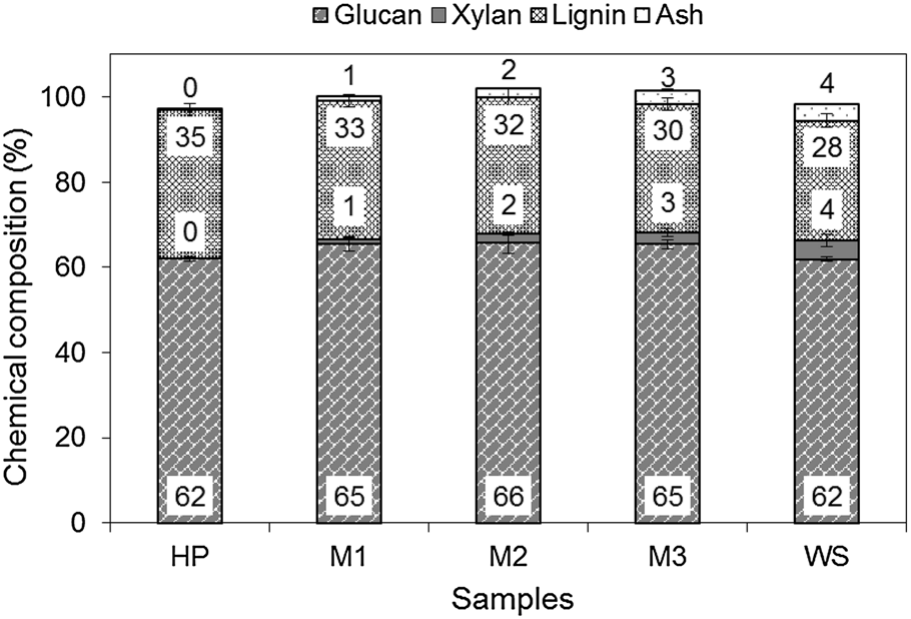
Composition of solid fractions of pretreated hybrid poplar (*HP*), mixtures (*M1*, *M2*, *M3*), and wheat straw (*WS*) expressed as % dry matter. *Values* and *error bars* represent the mean and the standard deviation from triplicate measurements

The chemical composition of solids after steam pretreatment, expressed as % dry matter for the different pretreated samples, is shown in Fig. 1. Only glucan, xylan, lignin, and ash were found in the resulting solids of the pretreated samples, ranging from 62 to 66 %, from 1 to 4 %, from 28 to 35 %, and from 1 to 4 %, respectively; except in HP, where xylan and ash were not found. At least 96 % of the hemicellulosic sugars were solubilized into the liquid fraction, except for minor quantities of WS xylan which was less labile than HP xylan. More xylose remained in solids of the mixtures containing more WS. Statistically significance differences were found among the samples in terms of solids ash content after pretreatment (*p* value <0.05). The ash content increased when more WS was added to the mixture since raw WS contained the highest ash content. Glucan and lignin comprised at least 90 % of the pretreated solids. No statistically significant differences were found for glucan content for all the samples. The lignin content in pretreated solids decreased when the proportion of WS increased even though the total lignin content was nearly identical among all the unpretreated samples. The total solids content after pretreatment was similar for all the samples ranging from 198 to 211 g (66 to 70 % solids recovery) (Additional file 1: Figure A1). The comparable chemical compositions after pretreatment of the different mixtures of biomass (M1–M3) demonstrate that steam pretreatment with SO_2_ is a fractionation method able to produce a homogeneous slurry from a diverse mixture of biomass containing different combinations of HP and WS.

### Liquid stream composition after pretreatment

The yields of sugars, acetic acid, furfural, 5-hydroxymethylfurfural (HMF), and total phenolics, and the pH in the pretreated liquid fractions were measured. Table 2 shows that glucose, xylose, and minor sugar yields, expressed as kg per tonne of raw biomass, ranged from 5 to 63 kg/tonne, from 75 to 146 kg/tonne, and from 6 to 13 kg/tonne, respectively. The majority of the sugars were in monomeric form, ranging from 67 to 98 %, 77 to 99 %, and 58 to 87 % for glucose, xylose, and minor sugars, respectively. The amount of glucose decreased with increased WS addition, which is consistent with the chemical composition of the raw material, where higher glucan content was observed in samples containing more HP. The amount of xylose solubilized in the liquid fraction was highest from feedstock with the highest fraction of WS, as expected, due to higher content of xylan in WS. The mixed biomass (M1–M3) showed the highest total sugar yield, ranging from 175 to 179 kg/tonne, demonstrating the benefits of mixing WS with HP feedstock for a lignocellulosic-based biorefinery.

**Table 2:** Monomeric sugar yield in liquid fraction of pretreated samples (kg/tonne of raw biomass)

	Glucose		Xylose		Minor sugars		Total sugars	
	Total	% mon^a^	Total	% mon^a^	Total	% mon^a^	Total	% mon^a^
HP	63 ± 3	93	75 ± 2	90	6 ± 1	72	144 ± 6	85
M1	59 ± 3	95	114 ± 4	99	6 ± 1	78	179 ± 8	91
M2	43 ± 3	98	119 ± 4	99	13 ± 2	87	175 ± 9	95
M3	20 ± 3	88	146 ± 6	99	11 ± 1	83	177 ± 9	90
WS	5 ± 1	67	116 ± 5	77	8 ± 1	58	129 ± 7	67

±Standard deviation are from triplicate measurements

*HP* hybrid poplar, *WS* wheat straw, *M1* 75 % HP, 25 % WS, *M2* 50 % HP, 50 % WS and *M3* 25 % HP, 75 % WS

^a^Monomeric sugar

Acetic acid, furfural from pentoses, HMF from hexoses, and phenolics from lignin were also found in the different liquid streams (Table 3). When more WS was added to the samples, less acetic acid and phenolics, and higher pH in the liquid fraction were found, ranging from 12 to 36 kg/tonne, from 30 to 34 kg/tonne, and from 1.4 to 1.8 pH, respectively. The yields of furfural and HMF for all the samples were less than 13 and 4 kg/tonne, respectively. These relatively low furan yields are due to the optimal steam pretreatment conditions which minimized sugar degradation. However, Rasmussen et al. [25] reported that various phenolics compound may also form as degradation products from glucose, xylose, and arabinose. In addition, recent progress has confirmed that carbohydrate degradation can form pseudo-lignin, which is an aromatic material that yields a positive Klason values and is not derived from native lignin [26]. Therefore, it might be possible that some of the sugars from HP, WS, and the different mixtures ended up as phenolics or part of a pseudo-lignin.

**Table 3:** Acetic acid, furans, phenolics yields (kg/tonne of raw biomass), and pH of the liquid fraction

	Acetic acid	Furfural	HMF	Total phenolics	pH
Hybrid poplar (HP)	36 ± 4	13 ± 1	4 ± 1	34 ± 3	1.4
M1 (75 % HP, 25 % WS)	31 ± 1	11 ± 1	2 ± 0	33 ± 4	1.5
M2 (50 % HP, 50 % WS)	29 ± 3	9 ± 1	2 ± 1	32 ± 1	1.6
M3 (25 % HP, 75 % WS)	21 ± 5	6 ± 2	1 ± 1	31 ± 4	1.7
Wheat straw (WS)	12 ± 4	4 ± 2	0 ± 0	30 ± 2	1.8

±Standard deviation are from triplicate measurements

The composition of the biomass mixture influenced the extent of hydrolysis of carbohydrates in the liquid stream. More monomeric sugars were found in the mixed biomass hydrolysate. Greater hydrolysis of hemicellulose in the mixed biomass may be explained by a mechanism of interaction between acetic acid and ash content in the mixed biomass samples. Acetic acid concentration increased by adding more HP, while ash content was increased by the addition of WS to the mixtures (Table 1). It is well known that during steam pretreatment of WS and HP, organic acids such as acetic acid, in conjunction with sulfur dioxide, generate acidic conditions to solubilize hemicellulosic sugar in the liquid stream. In this more acidic environment, more sugars are released in monomeric form but at the same time, more sugar degradation products are generated [18, 27]. The presence of ash, present at higher levels by adding WS to the mixtures, could ‘buffer’ extreme acidic conditions, avoiding the generation of sugar degradation products including furans. This synergistic effect, resulting from mixing HP and WS, is supported by the single HP and WS data. HP with the lowest pH (1.4) and the highest acetic acid content (3.7 %) contained more monomeric sugar (85 %) but higher furan content, indicating greater sugar degradation during pretreatment than with WS feedstock. WS had the highest pH (1.8) and ash content (4.2 %), contained only 58 % of the sugars in monomeric form, and has minimal furan formation.

The ash buffering effect could also be explained by differences in the buffering capacities of the different feedstocks and mixtures. High buffering capacity prevents acid production and makes the pretreatment appear less severe [28–30], generating less sugar degradation products. The buffering capacity was determined by hot water extraction and titration with 0.01 M H_2_SO_4_ (Method description, Additional file 2). Deionized water was used as a reference. The results show (Additional file 3: Figure A2) that initial pH values of water extracts from biomass were 5.03, 5.34, 5.71, 5.78, and 5.25 for HP, M1, M2, M3, and WS, respectively. Water extracted from single biomass was more acidic in comparison with the mixtures. Adding WS to the mixtures increased the pH of the biomass extracts. The titration showed a decreased from initial pH of water extracts to 3.0 in HP, M1, M2, M3, and WS after consumption of approximately 11, 22, 28, 50, and 41 ml of 0.01 M H_2_SO_4_, respectively. It is apparent that the buffer capacity increased when WS was supplemented to the mixtures.

Similarly, Harris et al. [31] observed that the presence of ash decreased the severity of dilute acid hydrolysis of red oak. They reported that there is an imbalance between inorganic cations and inorganic anions in biomass ash. Part of the cations in biomass therefore is probably bound to carboxylic acid group or is present as carbonates. The cations of weak acids salts are free to react with anions of stronger sulfuric acid and will partially neutralize it, thereby exerting a negative effect on the catalytic activity of the hydronium ions during pretreatment [28].

### Sugar recovery after steam pretreatment

Sugar recovery of monomeric glucose, xylose, and minor sugar was calculated for each of the samples after steam pretreatment. Table 4 shows the total sugar recovery expressed as % of the theoretical amount available in the raw material. For all samples, the sugar recovery ranged from 22 to 30 %. Nevertheless, statistically significant differences in recovery (*p* value <0.05) were found between mixed (M1, M2, and M3) and single biomasses (HP and WS), ranging from 29 to 30 % and from 22 to 23 %, respectively. The higher sugar recoveries observed for the HP and WS mixtures are a consequence of the improved hydrolysis—higher liquid phase sugar recovery—in the mixed samples. As result, the mixed biomass (M1, M2, and M3) showed a total monomeric sugar recovery on average 22 % higher than single biomass (HP and WS). Consequently, blending the two raw materials improved the monomeric sugar recovery after steam explosion.

**Table 4:** Monomeric sugar recovery after pretreatment (% of the theoretical available in the raw material)

Hybrid poplar (HP)	23.2 ± 1.0
M1 (75 % HP, 25 % WS)	29.6 ± 1.3
M2 (50 % HP, 50 % WS)	28.5 ± 1.5
M3 (25 % HP, 75 % WS)	29.2 ± 1.5
Wheat straw (WS)	22.1 ± 1.2

Sugar recovery considers monomeric glucose, xylose and minor sugars

±Standard deviation are from triplicate measurements

### Enzymatic hydrolysis

**Fig. 2:**
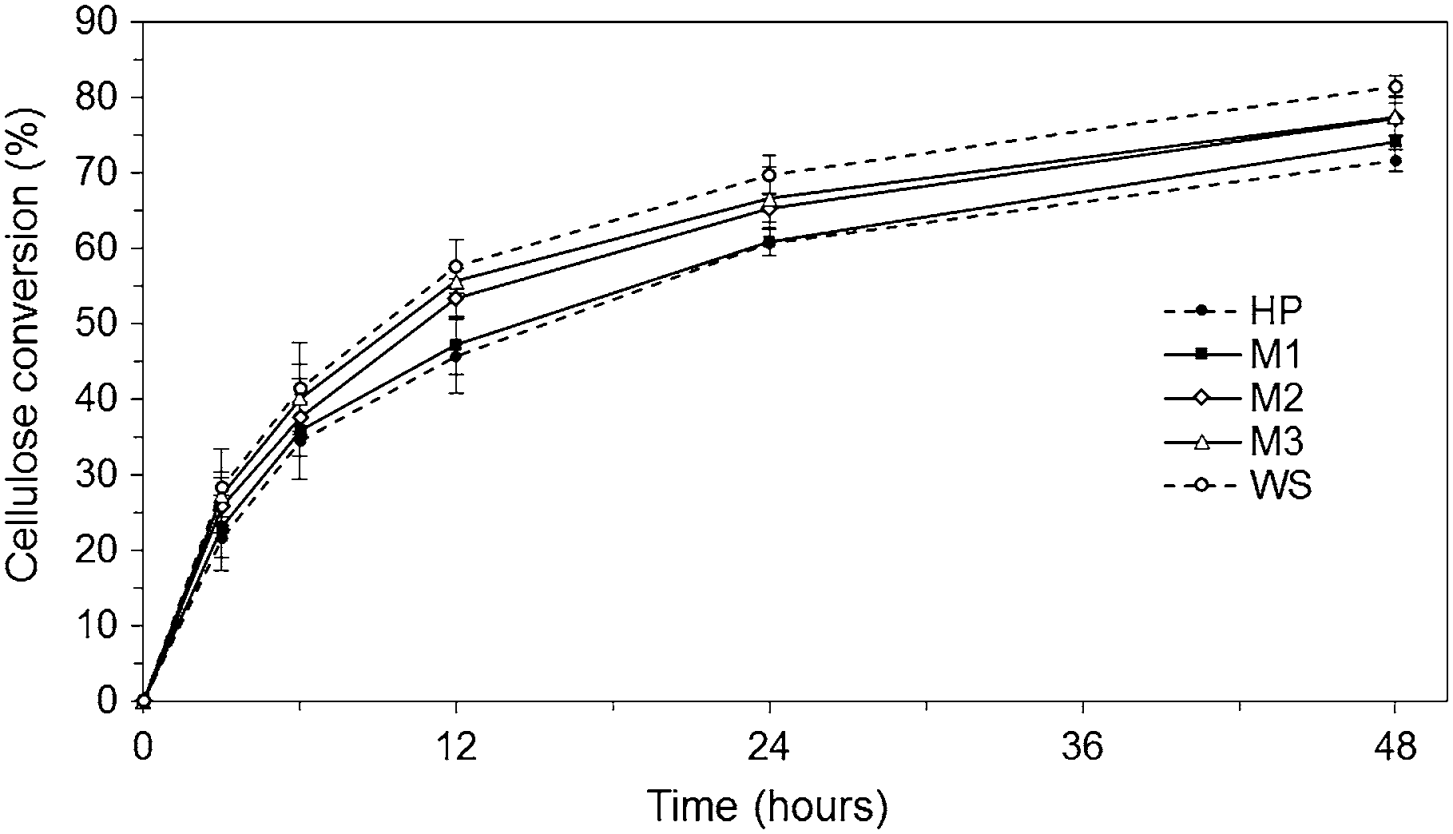
Cellulose to glucose conversion for steam pretreated hybrid poplar (*HP*), mixtures (*M1*, *M2*, *M3*), and wheat straw (WS) during enzymatic hydrolysis at 5 % (w/v) solids consistency and 5 FPU/g cellulose and 10 CBU/g cellulose enzymes loading. *Values* and *error bars* represent the mean and the standard deviation from triplicate measurements

The enzymatic digestibility of the washed pretreated solids was evaluated. All the samples were enzymatically hydrolyzed at 5 % consistency and 5 FPU/g cellulose enzyme loading. Figure 2 shows the cellulose to glucose conversion after 48 h of saccharification. The extent of cellulose conversion highlights the differences in digestibility between HP and WS, as well as the mixed biomass (M1, M2, and M3). For all samples, a conversion range from 73 to 81 % was observed after 48 h of enzymatic hydrolysis. A statistically significance difference was found between saccharification of HP and WS with a 73 and 81 % cellulose to glucose conversion, respectively (*p* value <0.05). Among the mixtures, conversions ranged from 74 to 77 % with no statistical differences.

It has been shown previously that WS is more responsive to hydrothermal pretreatment, resulting in a solid substrate that is easier to hydrolyze than poplar wood pretreated substrates [32]. Lignin content and lignin composition are among the key substrate components that control enzymatic hydrolysis efficiency [33, 34]. It is well known that woody biomass and grasses have different types of lignin. Poplar lignin is made up of only guaiacyl and syringyl units, while WS lignin contains guaiacyl, syringyl, and hydroxyphenyl units [35–37]. The lower lignin content and different lignin composition contribute to the improved saccharification of WS solids compared to HP.

### Total sugar yields

**Fig. 3:**
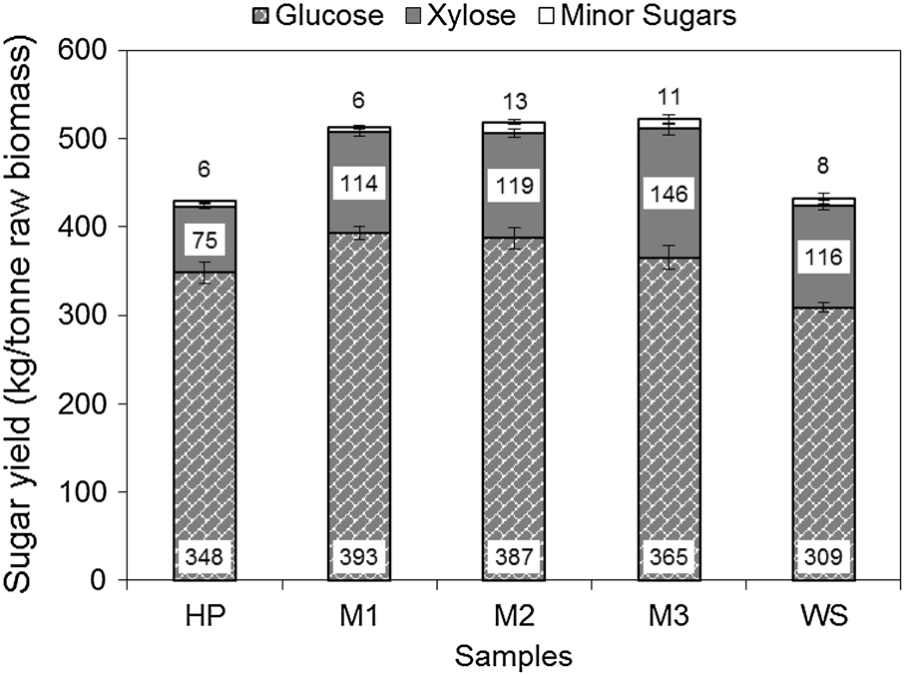
The overall sugar yields of glucose, xylose, and minor sugars after pretreatment and enzymatic hydrolysis in the solid and liquid fractions, expressed as kg of monomeric sugars per tonne of raw biomass. *HP* hybrid poplar, *M1*, *M2*, and *M3* mixed biomass, *WS* wheat straw. *Values* and *error bars* represent the mean and the standard deviation from triplicate measurements

The overall monomeric sugar yield following enzymatic hydrolysis was calculated for each single biomass (HP and WS) and the three different HP and WS mixtures (M1, M2, and M3). Figure 3 shows the overall sugar yields of glucose, xylose, and minor sugars (combined solid and liquid fractions) after pretreatment and saccharification, expressed as kg of monomeric sugar per tonne of raw biomass. Glucose, xylose, and minor sugars content ranged from 309 to 393 kg/tonne, from 75 to 146 kg/tonne, and from 6 to 13 kg/tonne, respectively. Only single biomass data found in the literature may be used for comparison, since no prior research has been done using mixtures containing HP and WS. Wyman et al. [38] steam pretreated and saccharified poplar wood using similar pretreatment conditions (3 % SO_2_, 190 °C, 5 min) but 3 times higher enzyme loading than in the present study. After pretreatment and saccharification, Wyman’s group [38] obtained 100 and 64 % of monomeric glucose and xylose recoveries, respectively. In the current study, the glucose and xylose recoveries were 76 and 56 %, respectively. The lower recovery in our study is attributable to the low enzyme loading. The xylose recoveries after pretreatment, however, were nearly identical in the two studies: 54 % in Wyman’s group and 56 % in our investigation (data not shown).

For WS, Linde et al. [39] steam pretreated 0.2 % H_2_SO_4_-impregnated WS at 190 °C for 5 min, resulting in 3.7 and 73.5 % of glucose and xylose recoveries after pretreatment (monomers plus oligomers). In the present study, after steam explosion, similar recoveries were observed: 2.1 % for glucose and 77.1 % for xylose (data not shown). The overall sugar recovery (glucose plus xylose monomers) from WS after pretreatment and enzymatic hydrolysis reported by Linde et al. [39] was similar to that obtained in the present study: 69 % from Linde et al. [39] compared with 74 % in the present study.

In this research, we found similar overall monomeric sugar yields from both HP and WS. The HP has a higher glucan content but the WS had slightly more xylan and liberated more monomeric sugars in both pretreatment and enzymatic hydrolysis. The net result was equal to overall sugar yields: 429 kg/tonne for HP and 432 kg/tonne for WS.

Statistically significance differences were found between single biomass and the mixtures (*p* value <0.05). The mixtures had high sugar yields ranging from 513 to 521 kg/tonne of biomass, 20 % greater than that obtained from the single biomass feedstock. Higher glucose, xylose, and minor sugar yields after pretreatment and saccharification were found by mixing HP and WS. This trend can be explained by the synergistic effect observed by blending these two types of biomass during pretreatment. Specifically, a mechanism of interaction between acetic acid in HP and ash in WS improved hemicellulose solubilization and reduced sugar degradation. HP saccharification also was improved by adding WS. Consequently, the sugar yields net result was remarkably higher for the mixtures.

Based on this research results, it is evident that processing mixed feedstocks containing different combination of HP and WS will improve the sugar yields after steam pretreatment and enzymatic hydrolysis. Mixing lignocellulosic biomass provides an opportunity to increase the productivity and profitability of biorefineries located in the Pacific Northwest where both feedstocks will be available. Use of mixed biomass also has the benefits of eliminating disruptive single feedstock campaign runs and will enable use of higher ash materials like WS that might otherwise result in equipment fouling or abrasion difficulties. Future techno-economic analysis and life cycle assessment will better define the benefits of using mixed poplar and WS feedstocks in a biorefinery.

## Conclusions

In this study, the impact of using mixed poplar and WS biomass feedstocks on overall sugar yields from a bioconversion process was assessed. It was found that mixing these two types of biomass positively affects the bioconversion process. Mixed biomass exhibited on average 20 % more sugar production than either single biomass. It was postulated that there is a synergistic effect caused by interaction of acetic acid in HP and ash in WS that results in more hemicellulose solubilization and less sugar degradation during pretreatment. Good sugar yields were obtained with all the samples, demonstrating that SO_2_-catalyzed steam pretreatment is a robust and efficient fractionation process that can readily process mixed feedstocks. Mixing of HP and WS will enable biorefineries to minimize their raw materials costs and provide an excellent opportunity for increasing the productivity and profitability in cellulosic biorefineries.

## Methods

HP, WS, and three mixtures with different combinations of both types of biomass (M1: 75 % HP, 25 % WS; M2: 50 % HP, 50 % WS and M3: 25 % HP, 75 % WS) were impregnated with SO_2_ (3 % w/w), steam pretreated at 195 °C for 5 min and enzymatically saccharified at 5 % (w/v) solids consistency, 5 FPU/g cellulase to determine the influence of using mixed biomass on overall sugar yields via bioconversion. A complete mass balance of carbohydrates was determined to assess overall sugars yield.

### Materials

Two types of biomass were used for this study. Freshly harvested 18-year-old HP, *Populus deltoides* × *Populus nigra*, from Puyallup, WA, USA was kindly provided by Washington State University and WS, *Tritricum* spp. from Eastern Washington, was kindly provided by the Science Center of the University of Washington. HP was comminuted with a slant disk chipper (Acrowood, Everett, WA) and then screened to particles approximately 2 cm × 2 cm × 0.4 cm with a moisture content of 60 %. WS was baled and received as full length straw. Straw was then chipped with an ECHO Bear Cat Chipper/Shredder to straws less than 2–3 cm with a moisture content of 10 %. Prior to pretreatment, each type of biomass was submerged in water for 24 h. Each biomass was then centrifuged to remove the excess water to obtain a moisture content of 60 %. HP and WS were used to prepare three different mixtures containing different proportions of each type of biomass (M1: 75 % HP, 25 % WS; AM2: 50 % HP, 50 % WS, and M3: 25 % HP, 75 % WS).

### Calculation of sugar recovery

Material balances for each biomass were closed for steam pretreatment and enzymatic hydrolysis by measuring the composition and total mass of each liquid and solid stream leaving pretreatment and saccharification and converting these data to amount of sugars recovered. The calculations were based on Wyman et al. [38]. Recoveries were then calculated based on glucose, xylose, and minor sugars available in the raw material fed to the systems. Thus, based on HP composition and the appropriate increase in mass with hydrolysis, a maximum of 461.1, 133.3, and 25.6 mass units of glucose, xylose, and minor sugars, respectively, could be produced from 1000 mass units of baseline HP biomass for a total maximum sugar potential of 620 mass units per 1000 units of dry poplar. Thus, the maximum possible total sugar recovery is 62 %. Identical procedures were completed for WS and the mixtures.

### Pretreatment and processing conditions

Prior to pretreatment, 300 g of dry biomass of each of the five feedstocks (HP, M1, M2, M3, and WS) was impregnated with gaseous sulfur dioxide (3 % w/w) and sealed in airtight plastic bags. Specifically, 9 g of SO_2_ was added to the 300 g of dry biomass from a gas cylinder into a plastic bag containing the biomass. The weight of the biomass was monitored overnight to determine the gas retention for each feedstock. Samples were then subdivided into 150 g samples and pretreated using a 2.7 l batch steam gun manufactured by Aurora Technical (Savona, BC, Canada) at 195 °C for 5 min. At the end of the reaction time, a pneumatic valve was opened between the pressurized reaction tank and the collection tank, causing the explosion of the biomass, which was discharged into the collection tank. The resulting slurry was vacuum filtered to separate the liquid fraction from the pretreated solids. Both fractions were analyzed as described below and used to construct a mass balance of carbohydrates and lignin. Solids were washed with deionized water equal to 20 times the mass of solids prior to analysis and hydrolysis. Each steam explosion was performed 3 times for each sample, resulting in a total of 15 substrates, all of which were analyzed as described below.

### Instrumental analysis

#### High-pressure liquid chromatography (HPLC)

Carbohydrates were measured on a Dionex (Sunnyvale, CA, USA) HPLC (ICS-3000) system equipped with an autosampler, electrochemical detector, dual pumps, and anion exchange column (Dionex, CarboPac PA1). Deionized water at 1 ml/min was used as an eluent, and post-column addition of 0.2 M NaOH at a flow rate of 0.5 ml/min ensured optimization of baseline stability and detector sensitivity. After each analysis, the column was reconditioned with 0.25 M NaOH. Twenty microliters of each sample were injected after filtration through a 0.22-µm syringe filter (Restek Corp., Bellefonte, PA, USA). Samples were measured against standards consisting of arabinose, galactose, glucose, xylose, and mannose. In addition, fucose was used as an internal standard.

Acetic acid, furfural, and HMF were measured using refractive index detection on a Shimadzu Prominence LC. Separation of these compounds was achieved by an anion exchange column [REZEX RHM-Mono-saccharide H^+^ (8 %), Phenomenex, Inc., Torrance, CA, USA] with an isocratic mobile phase that of 5 mM H_2_SO_4_ at a flow rate of 0.6 ml/min. The column oven temperature was maintained at 63 °C. Twenty microliters of each sample were injected after being appropriately diluted in deionized water and filtered through a 0.22-µm syringe filter (Restek Corp., Bellefonte, PA, USA). Standards were prepared and used to quantify the unknown samples.

### Compositional analysis

#### Ash and extractives

Ash content of raw biomass samples was measured gravimetrically by heating 20-mesh-milled dry biomass to 550 °C for 20 h using NREL protocol [40]. Water and ethanol extractives of raw biomass were determined according to NREL methods [41].

#### Insoluble carbohydrates, acetate groups, and lignin

Raw materials as HP, WS, and the mixtures (M1, M2, and M3) and respective pretreated solids were analyzed gravimetrically for lignin content, photometrically for soluble lignin, and by HPLC for carbohydrate and acetates content according to NREL protocols [42–45]. Briefly, 0.2 g of finely ground oven-dried sample was treated with 3 ml of H_2_SO_4_ at 72 % for 120 min at room temperature, then diluted into 120 ml total volume, and then autoclaved at 121 °C for 60 min. The autoclaved samples were then filtered using a glass crucible to separate the AIL from the ASL and carbohydrates. The AIL was determined by weighting the oven-dried crucibles, and the ASL in the filtrate was analyzed by ultraviolet absorbance at 205 nm. The filtrate was analyzed by HPLC for carbohydrate composition and acetic acid content.

#### Soluble carbohydrates

Monomeric and oligomeric soluble carbohydrates were determined using NREL LAP TP-510-42623 [46]. Briefly, 0.7 ml of 70 % H_2_SO_4_ was added to 15 ml of the liquid samples, and the volume made up to 20 ml with water. Samples were autoclaved at 121 °C for 60 min and analyzed by HPLC as described previously. Monomeric sugars were determined by analyzing the original samples without acid hydrolysis. Oligomeric sugar was calculated by subtracting monomeric sugar content from total sugar content determined after acid hydrolysis.

#### Total phenolics

Total phenolic concentration in the hydrolysates was assayed spectrophotometrically at 765 nm by Folin Ciocalteu method [47] using a FTIR spectrophotometer (Shimadzu, Tokyo, Japan). Gallic acid was used as calibration standard.

### Enzymatic hydrolysis

Enzymatic hydrolysis of washed solids was done at 5 % w/v solids in a total volume of 50 ml in 125-ml Erlenmeyer flasks. The solution was buffered at pH 4.8 with 0.05 M citric acid buffer, and the hydrolysis was carried out at 50 °C and 150 rpm shaking on an orbital shaking incubator (New Brunswick). Cellulase (Celluclast 1.5 L, 26 FPU/ml, Sigma) was added at 5 FPU/g cellulose, and supplemental beta-glucosidase (Novozym 188, 492 CBU/ml, Sigma) was added at 10 CBU/g cellulose. 1 ml samples were periodically removed and analyzed for glucose and xylose contents. Cellulase enzyme activity was determined using the bicinchoninic acid assay (BCA) based on Johnston et al. [48], and Kenealy and Jeffries [49]. The method is similar to filter paper assay (FPA), but involved the utilization of BCA as reagent, rather than dinitrosalicylic acid reagent (DNS). Cellobiase activity was determined by HPLC analysis.

### Statistical analysis

One-way ANOVA at *α* = 5 % was performed for each dependent variable to assess statistical differences as a function of the feedstock. In the cases where statistically significant differences were found (*p* value <0.05), a “Tukey test” (multiple comparison of means) was performed to find the specific group or groups of feedstock that was/were causing those differences. Data analysis was done using the statistical software R version 2.12.1

## Additional files

10.1186/s13068-015-0414-9 Total solids content after steam pretreatment for hybrid poplar (HP), mixtures (M1, M2, M3) and wheat straw (WS). Values and error bars represent the mean and the standard deviation from triplicate measurements.
10.1186/s13068-015-0414-9 Buffer capacity methodology of raw material.
10.1186/s13068-015-0414-9 Titration curves with 0.01 M H2SO4 for wheat straw (WS), hybrid poplar (HP) and the different mixtures (M1, M2, M3) extracts.

## Abbreviations

HP: Hybrid poplar
WS: Wheat straw
HMF: 5-hydroxymethylfurfural
FPU: Filter paper unit
HPLC: High-pressure liquid chromatography
AIL: Acid-insoluble lignin
ASL: Acid-soluble lignin
RPM: Revolutions per minute
CBU: Cellobiase unit
NREL: National Renewable Energy Laboratory
BCA: Bicinchoninic acid assay
FPA: Filter paper assay
DNS: Dinitrosalicylic acid
ANOVA: Analysis of variance
