# Supplementary material for: Synergistic effects of mixing hybrid poplar and wheat straw biomass for bioconversion processes
Source: Biotechnol Biofuels. 2015 Dec 24;8:226. doi: 10.1186/s13068-015-0414-9 (PMC4690274; doi:10.1186/s13068-015-0414-9)
Supplement: Supplementary file 1 — 10.1186/s13068-015-0414-9 Total solids content after steam pretreatment for hybrid poplar (HP), mixtures (M1, M2, M3) and wheat straw (WS). Values and error bars represent the mean and the standard deviation from triplicate measurements. [file 13068_2015_414_MOESM1_ESM.pdf]

Additional file A1

File name: Additional file A1

File format: PDF

Title of data: Solids

Description of data: Solids content after steam pretreatment

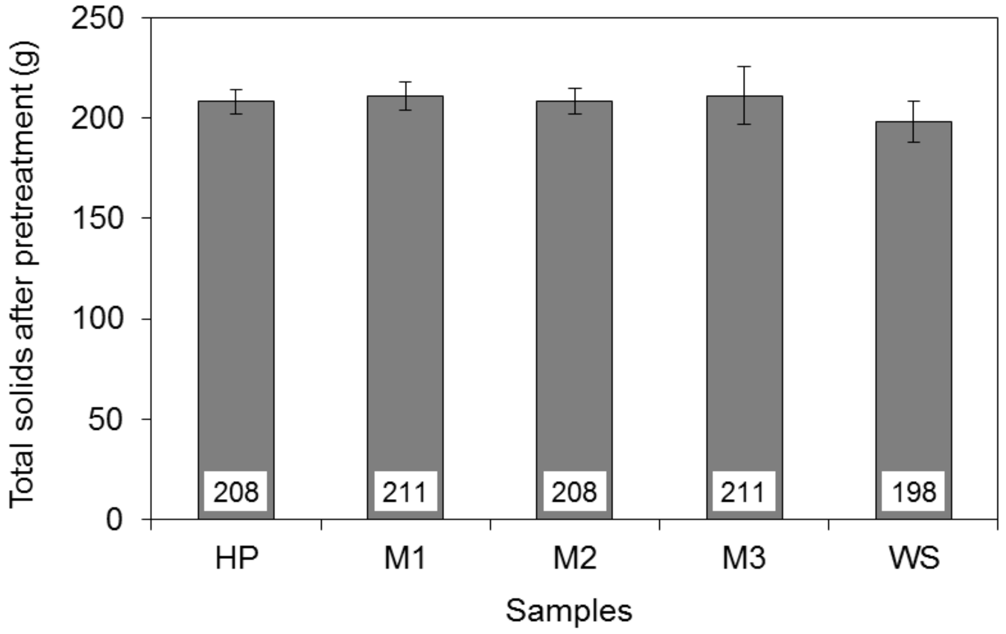

Figure A1. Total solids content after steam pretreatment for hybrid poplar (HP), mixtures (M1, M2, M3) and wheat straw (WS). Values and error bars represent the mean and the standard deviation from triplicate measurements
