# Supplementary material for: Synergistic effects of mixing hybrid poplar and wheat straw biomass for bioconversion processes
Source: Biotechnol Biofuels. 2015 Dec 24;8:226. doi: 10.1186/s13068-015-0414-9 (PMC4690274; doi:10.1186/s13068-015-0414-9)
Supplement: Supplementary file 2 — 10.1186/s13068-015-0414-9 Buffer capacity methodology of raw material. [file 13068_2015_414_MOESM2_ESM.pdf]

Additional file A2

File name: Additional file A2

File format: PDF

Title of data: Buffer capacity methodology

Description of data: Buffer capacity methodology of raw material

Buffering capacity methodology

To evaluate the buffering capacity of the raw material, 50 g of dry material of each feedstock was soaked in 1L deionized water at 80 °C for 30 min. The solid material was removed by filtration and the liquid was titrated with 0.01 M H_2SO_4. Distilled water was used as a reference solution [29].
