# Supplementary material for: Synergistic effects of mixing hybrid poplar and wheat straw biomass for bioconversion processes
Source: Biotechnol Biofuels. 2015 Dec 24;8:226. doi: 10.1186/s13068-015-0414-9 (PMC4690274; doi:10.1186/s13068-015-0414-9)
Supplement: Supplementary file 3 — 10.1186/s13068-015-0414-9 Titration curves with 0.01 M H2SO4 for wheat straw (WS), hybrid poplar (HP) and the different mixtures (M1, M2, M3) extracts. [file 13068_2015_414_MOESM3_ESM.pdf]

Additional file A3

File name: Additional file A3

File format: PDF

Title of data: Buffer capacity figure

Description of data: Titration water extracts from raw material

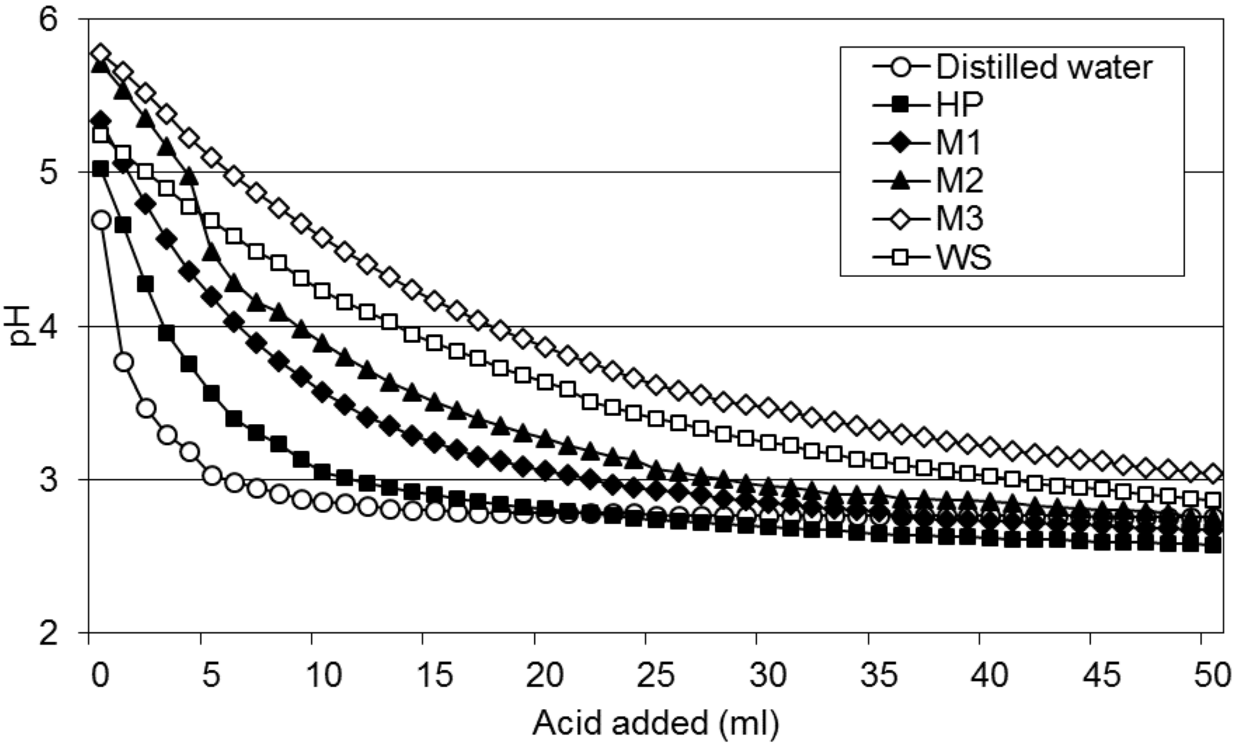

Figure A2. Titration curves with 0.01 M H_2SO_4 for wheat straw (WS), hybrid poplar (HP) and the different mixtures (M1, M2, M3) extracts
